# Vulvar Dermatofibrosarcoma protuberance in Iran and a narrative review of literature: A first case report

**DOI:** 10.22088/cjim.14.3.572

**Published:** 2023

**Authors:** Maliheh Arab, Nafiseh Faghih, Mahsa Asghari, Mona Agha Majidi, Behnaz Ghavami, Shahla Noori Ardebili

**Affiliations:** 1 Department of Gyneco-Oncology, Imam Hossein Medical Center, Shahid Beheshti University of Medical Sciences, Tehran, Iran; 2 Department of Gyneco-Oncology Taleghani Medical Center, Shahid Beheshti University of Medical Sciences and Health Services, Tehran, Iran; 3 Shahid Beheshti University of Medical Sciences. Tehran; 4 Tehran University of Medical Sciences Tehran, Iran; 5 Atieh Hospital, Tehran, Iran

**Keywords:** Dermatofibrosarcoma Protuberan, Fibrosarcomatous, Vulva, Neoplasia

## Abstract

**Background:**

Dermatofibrosarcoma protuberans (DFSP) is a soft tissue tumor that originates from deep layers of the dermis and mainly is common in young adults to middle age. This tumor is rare in the vulva.

**Case Presentation:**

A 53-year-old multiparous menopause female had complained of asymptomatic swelling of the right labia major. Dermatofibrosarcoma protuberans was reported in primary tumor resection. Six months later, rapid growing mass recurred in the vulva. The patient underwent radical Vulvectomy and resection of the margin of about 2-3 cm along with bilateral Inguinofemoral lymphadenectomy. The margins of the mass were negative in the frozen section. Microscopic examination revealed that hypercellular neoplasm in dermis comprising monomorphic spindle cells with high mitotic activity, some hyperchromatic nuclei arranged in palisading fashion. Microscopic and IHC study confirmed the conversion of dermatofibrosarcoma protuberans to fibrosarcoma.

**Conclusion:**

This case was presented due to the rarity of dermatofibrosarcoma protuberance in the vulva and pathologic conversion to fibrosarcoma.

Dermatofibrosarcoma protuberans (DFSP) is a soft tissue tumor that originates from deep layers of the dermis and mainly involves the trunk and proximal extremities in both sexes and is common in young adults to middle age (20-50 years). This tumor is rare in the vulva and tends to be localized. The vulvar tumor is usually present as a mass with different sizes in different parts of the vulva at any age. In terms of grading, they are usually included in the low to intermediate grade due to the infiltrative nature, especially in cases which a proper margin is not achieved in surgery, there is usually tumor relapse (1,2). 

## Case Presentation

A 53-year-old multiparous female (gravida 5 para 5 by normal vaginal delivery), mother of 5 children menopause for at least seven years living in Hamadan, located in the western part of Iran. She had complained of asymptomatic swelling of the right labia major. She was referred to a gynecologist due to a painless mass with a gradual increase in size. She was sexually active. Her family history was negative regarding cancer. She had no comorbidity or drug consumption other than vitamins. She operated on with impression of Bartholin mass. A mass of about 6 × 6 × 3 cm size was removed. The pathology report of the specimen was dermatofibrosarcoma protuberans (DFSP) and the margin of the lesion was positive. 

**Figure 1:**
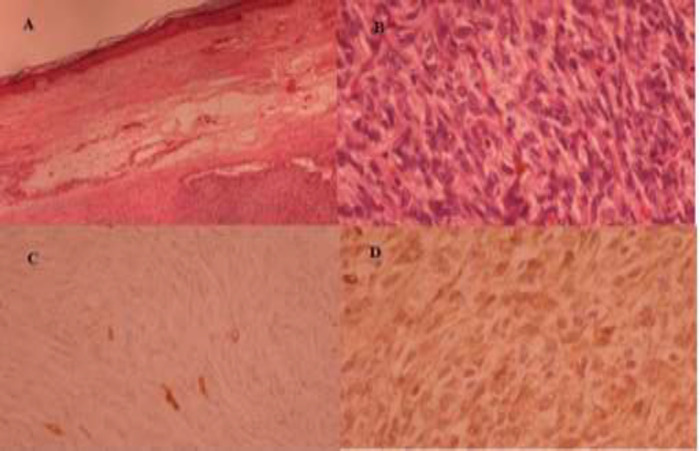
A: Storiform growth of a spindle cell tumor. B: The pleomorphic is mild to moderate with scant mitotic count (obj. 400), C: The tumor showing a low proliferation rate, Ki67 up to 1%. (obj.400), D: Immunohistochemistry staining showing diffuse CD34 positivity. (obj. 400)

In immunohistochemical study, CD34 and K167 were positive, which was consistent with the diagnosis of dermatofibrosarcoma. The patient, after six months of the first operation, returned with pain and rapidly growing mass in the vulva. She was referred to our center as the tertiary level center of gyneco-oncology. Clinical exam revealed a non-tender mass with a rigid consistency of about 7 × 8 cm in the right labia major with clitoral involvement and separate from the urethra. The skin covering the mass was intact. In the review of the slides and completion of IHC in our center, dermatofibrosarcoma protuberans was confirmed (figure 1).

**Figure 2:**
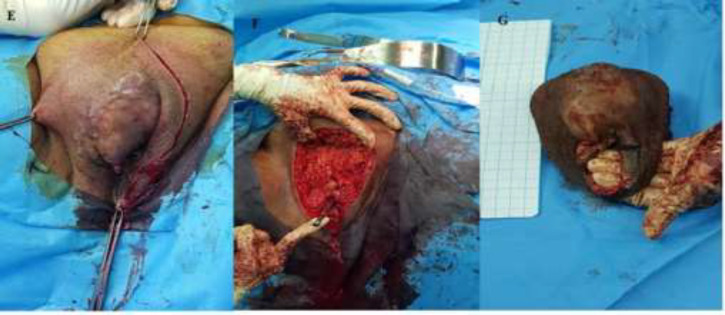
E: Macroscopic view of vulvar mass, F: Vulvar specimen after surgery, G: Resection site of vulvar mass

**Figure 3:**
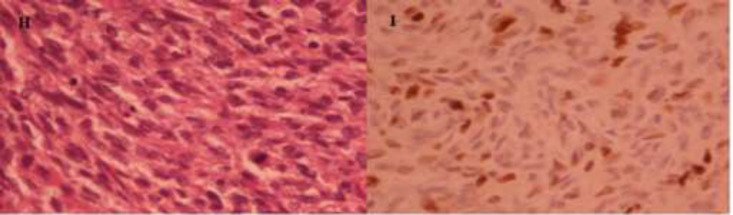
H: The tumor converted to Fibrosarcoma showing moderate polymorphism with a noticeable number of mitosis. (obj. 400), I: Ki67 immunostaining showing 30% proliferation rate. (obj. 400)

History, physical exam and pathologic report of the case presented in the joint clinic of gyneco-oncology for decision making and management, as the routine of cancer patients in Imam Hossein Medical Center. The patient underwent radical vulvectomy and resection of the margin of about 2-3 cm along with bilateral inguinofemoral lymphadenectomy due to deep palpation of inguinal lymph nodes. The margins of the mass lateral and deeply were negative in the frozen section. Morphology of the mass and removed specimen are shown in the images. The macroscopic specimen of the mass was 5 × 7 × 10 cm in size (figure 2). According to Musculoskeletal Tumor Society (MSTS) staging system for sarcomas, this patient was in stage IA and based on TNM system was in T2N0M0. Microscopic examination revealed: Hypercellular neoplasm in dermis comprising monomorphic spindle cells with high mitotic activity, some hyperchromatic nuclei arranged in palisading fashion. Microscopic and IHC study confirmed the conversion of dermatofibrosarcoma protuberans to fibrosarcoma (figure 3). In 18 months follow-up, the site of surgery is normal.

## Discussion

This is a case report and a narrative review, the search method is the word "dermatofibrosarcoma protuberans AND treatment" was searched in PubMed, Clinical Key databases and up to date in English articles without time limitations. 47 articles were extracted and 9 articles were selected after the initial review. Epidemiology: DFSP is a rare skin and soft tissue disease that is low to intermediate grade and often occurs in the trunk, proximal extremities, head, and neck. Vulvar origin is particularly rare.

This soft tissue sarcoma was first described in 1924 and its annual incidence is 4.2 per million or 0.1% of all cancers. It usually occurs in the 4th or 5th decade of life. In the black race is more common compared to the white. Although there is no sex difference. The DFSP has an infiltrative nature and is usually referred to as a tumor, a nodule or plaque that lies deep in the dermis layer. Typically, the degree of intermediate to low grade tends to have a large local recurrence and frequent recurrences of the tumor increase the risk of becoming more malignant types. Metastasis is rare in this type of tumor, some studies have reported metastasis rate about 1 to 5 % (2,3,4). Clinical Signs: Most patients present at about 42 years with vulvar mass which might be located occur in any part of the perineum. Mostly is located in the labia major area, and the left side is more common than the right side, with different size from 1.5 to 15 cm. The average size is 4 cm and the mass is usually painless. Even at large sizes, only the ambiguous perineal pain is felt due to pressure on the surrounding tissues. Usually, it grows slowly, and the patient ignores it, in examination, it is mistaken to Bartholin or Cebas cyst (1,2,5).

Pathology Diagnosis: In more than 90% of DFSP cases, unbalanced chromosomal translocation t [17, 22] occurs in a pathologic study of the monomorphic proliferation of Bland spindle cells with large nucleus and small cytoplasm that has a honeycomb appearance and subcutaneous fat infiltrate, in IHC study, CD34 and Vimentin are strongly positive, but S100 and factor XIII and the smooth muscle actin, Desmin, Keratin, and epithelial membrane antigen are negative (6,7).Cytological atypia and increased mitotic activity are not normally seen unless transformation to the fibrosarcoma occurs, as in the present case (2,4,3). Imaging: Confirmation of DFSP, by Core needle or excisional biopsy, is needed for a definite diagnosis. Palpation alone is not able to identify the depth of penetration, So MRI is more sensitive. Although in some locations, such as the head, neck, and extremities, detection of invasion to the surrounding tissues is difficult even with MRI (2). MRI is used to detect recurrence of the disease with a sensitivity of 60% and specificity of 100% if positive surgical margin is reported, MRI is not helpful to determine the amount of resident tumor. CT scan is used in very rare cases suspicious of bone involvement (2). 

**Treatment:**

Vulvectomy: The main treatment of DFSP is surgical removal of affected tissue through Wide Local Excision to radical Vulvectomy. The main point of surgery is about 3 centimeters negative margin. The widely negative margin patients are less likely to locally be recurred.

In some cases, the free margin will not be achieved because of the anatomical site where it is not possible or in the case of post-operation diagnosis, in which case the incidence of relapse is higher (2,8). The best treatment for DFSP is the use of the Mohs Microscopic Surgery (MMS) procedure, which requires a skilled surgeon and nurse to carefully remove tissues from margin areas and then fix them. So, there is a possibility of accurate examination of the margin and in some cases, IHC is also used during surgery. The lowest marginal negative marker required for DFSP control is not known, and in the mapping approach with MMS surgery, this margin is reported to be 3 cm. In some studies, this margin has also been reported to be 5cm. 

In cases where the lesion is large, or in cases of DFSP recurrence, it is better to use the MMS method, which uses horizontal cuts instead of removing vertical sections of the tissue. Immediately, the frozen is done on the tissue until the negative margin is reached and the CD34 is used on the frozen section. In the slow MMS method, the sample is usually fixed in formalin and then paraffinized to test for CD34, which is more reliable than the frozen section (9). Most of the studies that have been talked about MMS surgery are retrospective, and so far no randomized trial has been conducted to test this method better than other surgical procedures. The selection of MMS surgery or Wide Local Excision depends on the location, size of the tumor, risk of recurrence, the ability of the surgeon and its team to accomplish that surgical method, cost and cosmetic issues (2). 

Lymphadenectomy: The main DFSP surgery is the removal of lesions with negative margin to prevent local recurrence. Lymphatic involvement is very rare. Lymphadenectomy is not usually carried out in a prophylactic procedure. Only in cases of DFSP disease that lymphatic glands are involved, it is advisable to remove them (2,3). Radiotherapy and chemotherapy after surgery and complications: The benefits of adjuvant radiotherapy should be weighed against its complications. Due to the small number of DFSP cases, the data are collected from studies that have not been complicated. Anyway, in 6 patients with grade 2, fibrosis or telangiectasia of the radiotherapy site has been reported. In most studies, the use of postoperative radiotherapy has been advised, if the surgeon failed to reach enough margin and the possibility of recurrence is high, or in cases where the marginal tissue is positive and there is no possibility of re-resection and no plan of surgery in the future and in more than 5 cm size of the mass or in recurrence. (2,9,10). 

According to NCCN Guideline, radiotherapy is recommended only in cases where there is a large tumor or close or positive margin (9). There is little data on the use of chemotherapy in the treatment of DFSP, but it is suggested that the response rate of DFSP to the regimens used in the treatment of sarcoma is low. A case has been reported with advanced DFSP, which has responded to low-dose methotrexate and Vinblastin therapy (a regimen used for the treatment of Desmoids tumors) (2,9). 

Target Therapy: Reports used on Imatinib (orally active small molecule tyrosine kinase inhibitors), in patients with metastatic DFSP, have been shown to improve clinical response resulting from the approval of the treatment in patients with nonresectable recurrence and metastasis in Europe and the United States. Also, in patients who require radiotherapy after surgery due to close margins, but their wounds have complications and have not been recovered, this drug can be used. Also, Imatinib can be used as neo-adjuvant to reduce the size of the mass resulting in better cosmetic surgery. The duration of treatment and dosage of the treatment is not known. In general, the Imatinib as a standard treatment should not be used and should be limited to clinical trials to obtain more information (6,8,9).

Factors involved in the relapse of the disease and prevention of recurrence: The most important factor in the recurrence of DFSP is surgical margins. Generally, the chance of relapse is low in cases where the margin is negative (0-13%). However, in cases with positive or near margin, the relapse rate is between 21-82%. Some studies express that in cases with fibrosarcomatous changes, the chance of recurrence and metastasis increases (1,5,8). Distant metastasis through the blood is very rare and usually occurs in patients with multiple local recurrences and following inadequate primary tissue removal. The most commonly involved organ is usually the lung, but the metastasis of the brain, bone, and other soft tissues has been reported, as well (2,3). Prognosis: In general, the prognosis of DFSP is good, and mortality is rare. The complications of surgery include ulcerative infection and in case of insufficient tissue removal (positive margin), localized relapse of the disease is still a problem (2). 

It seems that age over 50 is associated with increased relapse rates and reduced patient survivals. Sarcomatosis changes in tissue are also considered as a cause of disease recurrence, but the size of the mass appears to have no effect on the relapse or the survival rate of the patient (2). Follow-up: Local recurrence appears to occur mostly in the first 3 years of follow-up. However, recurrence has been reported in 25-50% of cases after 5 years (2). Follow-up after treatment is recommended for a lifetime. The best follow-up method is not known, but every 6 months for the first 3-5 years and annual examination after 5 years is recommended. The patient's clinical examination should include a thorough inspection and palpation of the location of the scar, and the patient has been oriented for a regular examination of that place herself (2). DFSP Types: Differential types of dermatofibrosarcoma protuberans include:

1. Fibrosarcomatous DFSP: Commonly characterized by fusiform cell proliferation and scant cytoplasm, which exhibit higher mitotic and pleomorphism than classical DFSP, usually in IHC, CD34 is negative for non-differentiation of cells, and in women, it is more than males.
2. Pigmented DFSP (Bednar Tumor).
3. Myoid DFSP: Nodules composed of fibroblastic cells with cigarette nucleus and large eosinophil cytoplasm, which in IHC are usually SMA positive and Desmin negative. In this type of DFSP, CD34 may also be negative, and this type can also be seen in women.
4. Atrophic DFSP.
5. Myxoid DFSP: Typically, the branches of the thin-wall vessels and the stellate cells that are negative in S100 IHC.
6. Granular cell DFSP (3).

Differential Diagnosis: Different diagnoses include Cellular Dermatofibroma, Cellular Leiomyoma, Cellular Neurofibroma, Low-Grade Lymphosarcoma, Fibrosarcoma, Low-Grade Schwannoma, Desmoplastic Melanoma, which in some cases may help to diagnose the disease with the location of the tumor and associated IHC, but in some cases, the diagnosis has not been true after treatment and reassessment (1,5,11). This case was presented due to the rarity of Dermatofibrosarcoma protuberance in the vulva and pathologic conversion to Fibrosarcoma. Informed consent was obtained from the patient and this study complied with the Declaration of Helsinki Principles. Permission to undertake the study was obtained from the Ethics Committee of SBUMS.
